# Long-term temozolomide might be an optimal choice for patient with multifocal glioblastoma, especially with deep-seated structure involvement: a case report and literature review

**DOI:** 10.1186/s12957-015-0558-x

**Published:** 2015-04-09

**Authors:** Yunpeng Liu, Shuyu Hao, Lanbing Yu, Zhixian Gao

**Affiliations:** Department of Neurosurgery, Beijing Tiantan Hospital, Capital Medical University, No. 6 Tiantan Xili, Dongcheng District, Beijing, 100050 China; China National Clinical Research Center for Neurological Diseases, No. 6 Tiantan Xili, Dongcheng District, Beijing, China

**Keywords:** Multifocal glioblastoma, Deep-seated, Thalamus, Temozolomide, Long-term chemotherapy

## Abstract

**Background:**

Multifocal glioblastoma is an uncommon and refractory subtype of high-grade glioma since the burden of masses could not be eliminated simply by operation, and it is getting even harder to control if some deep structures, like thalamus and pineal region, are involved.

**Case presentation:**

Here we report a case of a 30-year-old male with multifocal glioblastoma affected his right thalamus, left lateral ventricle, and pineal region. Clinical manifestations include operation, concurrent radiochemotherapy, and a 12-cycle adjuvant temozolomide administration. The masses of this patient nearly disappeared after 15 months from the primary diagnosis, and no severe adverse event or neurological sequel occurred.

**Conclusions:**

Long-term temozolomide might be an optimal choice for patients with multifocal glioblastoma, especially with deep-seated structure involvement.

## Background

Glioblastoma multiforme (GBM) is the most common primary brain malignancy, and the median survival is less than 15 months [1]. Multifocal glioblastoma has been defined as glioblastoma being found synchronously in multiple foci, and there is a presumed microscopic connection [2]. Multifocal glioblastoma often has a worse prognosis than solitary ones. Several studies showed significantly lower median survival about 7.6 to 12 months of patients with newly diagnosed multifocal or multicentric glioblastoma compared to solitary ones [3-5], in spite of operation plus radio-chemotherapy being carried out.

While radiotherapy with concomitant and adjuvant temozolomide (6 cycles, 150 to 200 mg/m^2^/day) is the standard treatment after surgery in glioblastoma patients, several institutions have studied on prolonged administration of temozolomide and show increased survival periods of these patients [6-9]. However, if the lesions affect deep-seated structures, like thalamus or post-third-ventricle structures, the operation is getting harder and patients often have even worse outcomes. Here we present a case report of a patient’s well-being with multifocal glioblastoma of which the thalamus and pineal regions were involved, who had received prolonged temozolomide in addition to standard treatment and finally got a relatively optimistic prognosis. We reviewed related literatures, considering that long-term chemotherapy might be an option for treating patients with deep-seated multifocal glioblastoma.

## Case presentation

### First visit and examination

**Figure 1:**
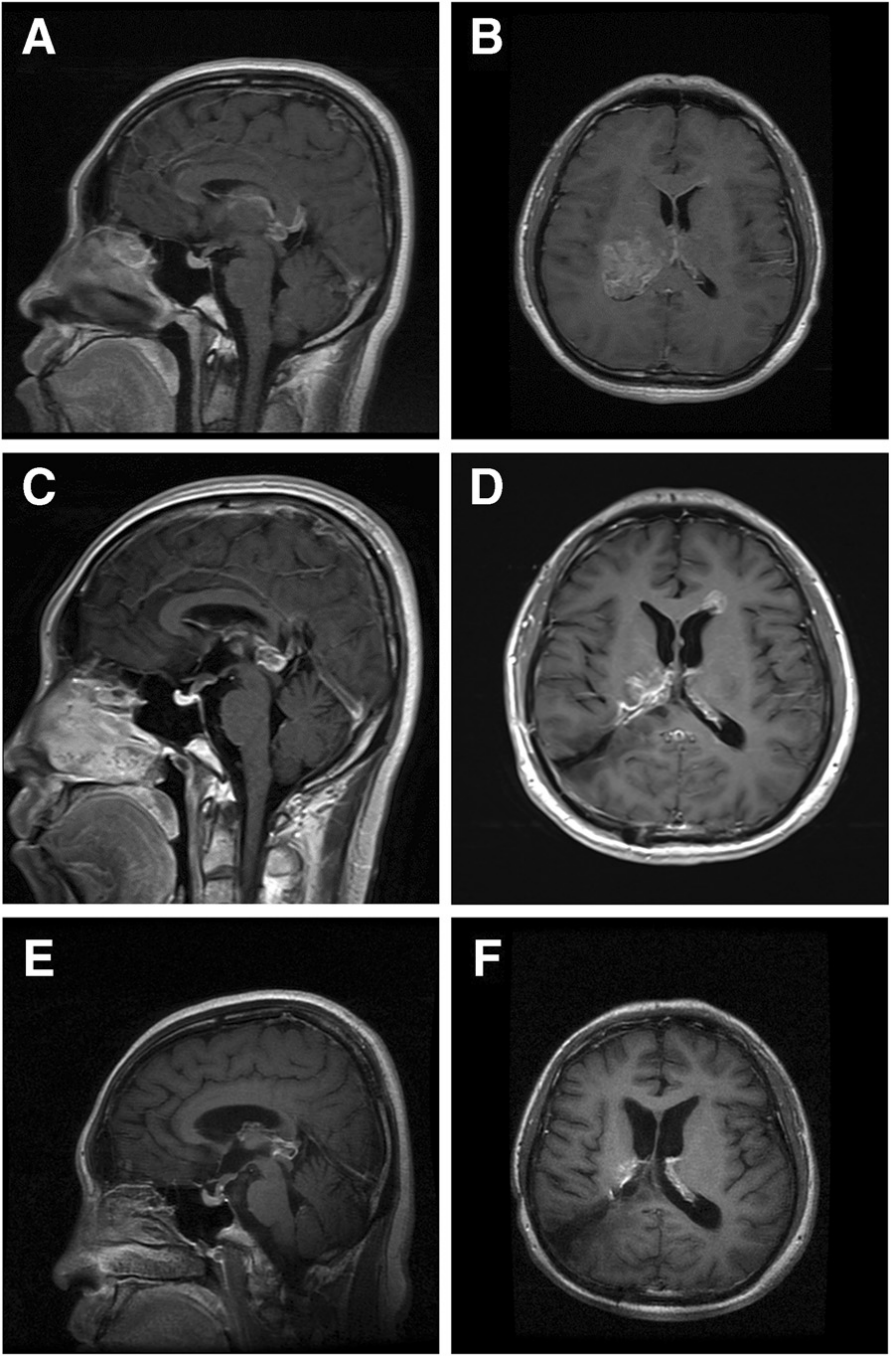
**The axial and sagittal contrast magnetic resonance imaging (MRI) results of the patient.** At primary diagnosis, there were enhancements of the right thalamus, anterior angle of left ventricle and pineal region **(A, B)**. After the operation, the lesion of the right thalamus was completely removed **(C, D)**. Lesions on the left ventricle and pineal region were eliminated after 12-cycle administration of temozolomide **(E, F)**.

A 30-year-old man with no significant past medical history or family history had felt numb of his left extremities for 1 month. He also complained of mild headache and interrupted vomiting. Physical examination at admission revealed no abnormal signs such as asymmetric pupils or hemiparesis. Cranial magnetic resonance imaging (MRI) showed multifocal lesions of the brain. The largest one was an occupying lesion at his right thalamus, with a compressive deformation of the right ventricle. This mass was mostly hyperintense on T1-weighted (T1W) and T2-weighted (T2W) images. After gadolinium (Gd) administration, the whole mass presented a heterogeneously enhanced character. While at the frontal angle of the left ventricle as well as pineal body region, two nodules with isointensity on both T1W and T2W images were detected, and they also mildly enhanced after Gd administration (Figure 1A).

### Initial resection

The patient underwent a right occipitotemporal craniotomy after admission. With ultrasound guidance, a 4-cm-deep colostomy was carried out through the patient’s supramarginal gyrus to the occipital angle of the right ventricle. The mass was seen at the lateral wall of the ventricle, with grey and red appearance, uneven texture, and rich blood supply. There was an obscure boundary between the mass and normal tissue aside, as the tumor showed an infiltrative pattern of growth. Resection within the mass had alleviated the pressure, thus releasing some cerebrospinal fluid, which lowered the pressure even more. Then a total resection of the mass around its approximate boundary was achieved (Figure 1B).

### Histopathological examination

**Figure 2:**
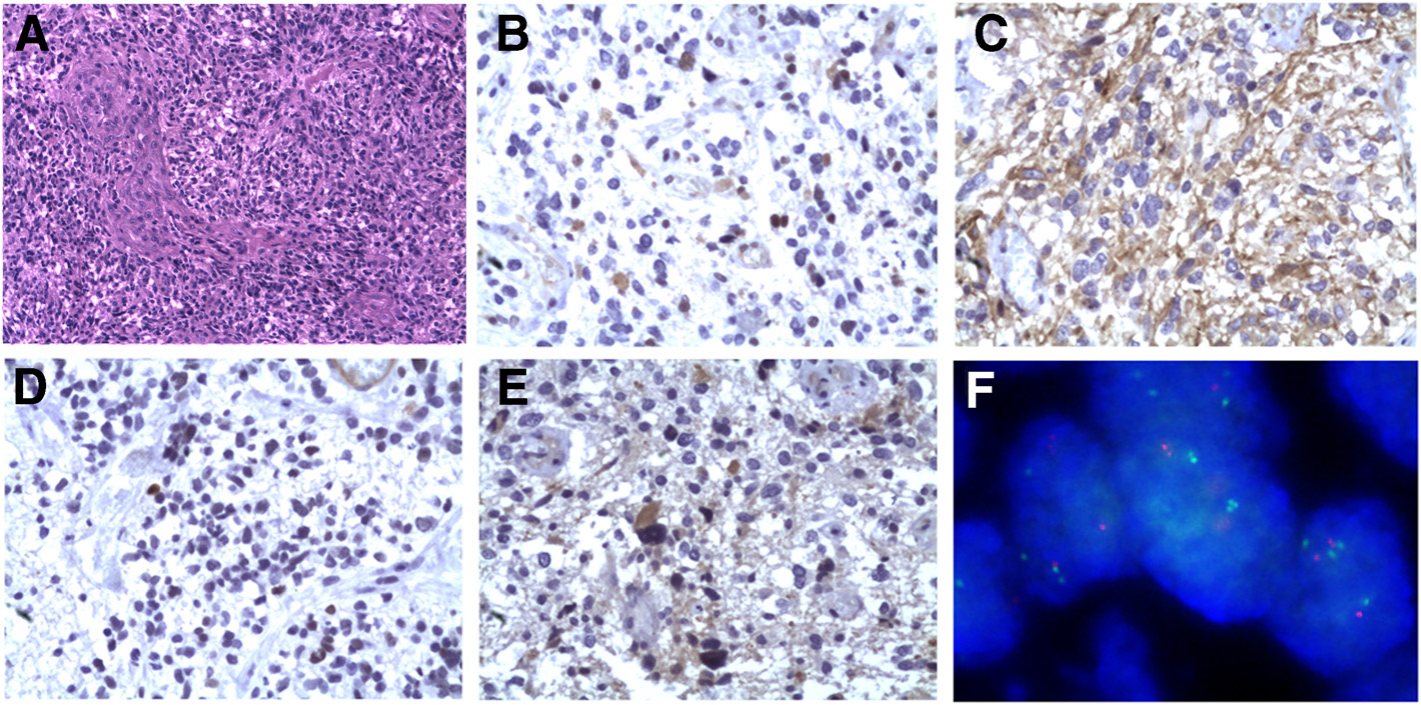
**Histopathology of the tumor of the patient.** A mount of large and bizarre giant cells compatible with a diagnosis of glioblastoma in the H-E staining **(A)**. Immunohistochemically, the MGMT promoter was weakly methylated **(B)** and strong mutation of PTEN genes **(C)**, P53 **(D)**, and amplificated VEGF genes **(E)** were seen. The immunofluorescent staining of 1p19q showed no deficiency existed **(F)**.

The examination was approved by the Institutional Review Board of Beijing Tiantan Hospital, Capital Medical University. On gross examination, the surgically excised tissue, with a volume of 5.0 × 5.0 × 4.0 cm^3^ approximately, was heterogeneously soft and rough with pinkish color. On microscopic examination, the tumor was composed of a mount of large and bizarre giant cells with abundant eosinophilic cytoplasm on hematoxylin-eosin (H-E) staining, which was compatible with a diagnosis of glioblastoma. Scattered cells with loosely cohesive cytoplasm (that is, ‘vacuole sign’) could be seen, which indicated the presence of an oligodendroglioma component (Figure 2A). Immunohistochemically, the astrocytic tumor cells showed weakly positive on the methylation of genes of O-6-methylguanine DNA methyltransferase (MGMT) promoter (Figure 2B) and strong mutation of phosphatase and tensin homolog (PTEN) genes (Figure 2C). Additionally, the tumor cells were both immunoreactive for P53 (Figure 2D) and had amplificated vascular endothelial growth factor (VEGF) genes (Figure 2E). The immunofluorescent staining of 1p19q showed there were no mutations existed (Figure 2F).

### Postoperative concurrent radiochemotherapy

After the resection, the patient presented with lucid mind but mental fatigue, his left extremities’ muscle strength is grade I, while his right ones’ is grade V. The patient left the neurosurgery department with a wheelchair, and his Karnofsky Performance Scale (KPS) score is 50. Concurrent radiotherapy and chemotherapy were administered 1 week later, and the radiotherapy was three-dimension conformal and intensity-modulated which covered both the left ventricle and the surgical cavity of the right thalamus, with a dosage of 58.0 Gy in 29 fractions. At the meantime, the patient had continuous temozolomide, 160 mg (75 mg/m^2^/day) per day for 42 days. During this adjuvant therapy, the patient’s mental status got normalized, and his left hemiparesis was gradually improved with grade-IV strength of the arm and grade-III strength of the leg at the end of the medication. Complications other than mild nausea did not occur.

### Long-term temozolomide therapy and subsequent visits

A follow-up MRI study performed 4 weeks after radiotherapy (14 weeks after resection) showed thickened enhancement after Gd administration in the pineal body region and the frontal angle of the left ventricle, compared with the pre-radiation MRI study, which might indicate tumor dissemination to these areas. Adjuvant temozolomide with 150 to 200 mg/m^2^ for 5 days every 28 days was then administered to the patient for 12 cycles. During the long-term therapy, the patient was followed by MRI and clinical visits every 2 to 4 months. Blood testing for hematologic toxicity was performed every cycle, and the results did not show abnormal blood cell counting or changes in liver and kidney functions. Gastrointestinal toxicity or allergy was also not seen. After 8 cycles, the MRI showed significantly weakened signal in the frontal angle of left ventricle as well as the pineal body region on the Gd enhanced images. At the end of the 12-cycle therapy, the enhancement area of left ventricle almost disappeared, the same change presented in the pineal body region as well (Figure 1C). At the recent clinical visit, the patient showed normal mental status, along with grade-IV muscle strength of his left extremities. The patient was back to work as a green worker, and his KPS score was improved to 90.

## Discussion

Multifocal glioblastoma is defined as intracranial GBM with multiple synchronous foci, and there is a known anatomical route for spread of diseases between lesions. It only consists of a small part of glioblastoma, with an incidence of 0.5% to 20% [7,10,11]. Thomas *et al*. reported that with the help of improved imaging technology, the incidence of multiple GBM would increase [12], but deep-seated multifocal GBM, with thalamus and post-third-ventricle structure involved, remains rare and few literatures have been published to explain this disease. The presented case was a young male with multiple lesions located at his right thalamus, the frontal angle of the left ventricle and the pineal region were also involved, which made this case even rarer since the tumors of the pineal region only consisted of 0.5% to 2% of all intracranial neoplasms in several researches [13-15]. The multiple lesions made the treatment very complicated since one operation was not enough. We chose the craniotomy of a right lateral ventricle approach to remove the thalamic lesion since it was the largest one, which caused the neurological focal signs. From the postoperative MRI, gross total resection was achieved of this single target.

Multifocal GBM has many ways to spread intracranially, such as through commissual pathways, cerebrospinal channels, blood vessels, or by local dissemination through satellite formation [12]. The patient’s GBM presented in this literature probably had spread by a way combined with transventricular and subependymal routes, which was an uncommon pathway described by Kyritsis *et al.* [16].

Several literatures mentioned that GBM contained cells propagated from astrocyte-like neural stem cells within the subventricular zone (SVZ), which was located just under the ependymal of the brain ventricles [17,18]. Lim *et al*. [19] pointed out that GBMs with contact to SVZ were most likely to be multifocal at diagnosis. We addressed that the thalamic mass, which caused the right ventricle compression of this patient, might had different cells of origin and, thus, was responsible for its metastatic behavior. Pathological examinations for this tumor revealed a PTEN-mutated, MGMT-methylated, and VEGF-amplificated condition. Many pathological researches revealed PTEN mutation was a common event of glioblastoma genesis [20-22]. Krex *et al*. [23] found nonsense mutation located in exon 7 in the cell lines of three multifocal GBM, and this mutation was identified in the original tumor tissue but not in the germ-line DNA, which suggested that the mutation was an early event of pathogenesis of the tumor before it separated into different lesions. Recently, Xu *et al*. [24] found that PTEN nonsense mutations increased genomic instability of GBM and shortened disease-free survival of GBM patients. On the other hand, some researches caught thoughts of brain tumor stem cells had higher expression of VEGF and, as a result, built a suitable niche for tumor proliferation [25,26]. For example, Bao *et al*. found that bevacizumab, a well known antiangiogenic agent, suppressed growth of stem cell-like glioma cells but limited efficacy against those derived from non-stem cell-like glioma cells *in vivo* [27] and, thus, might give an explanation to the different prognosis among patients under the treatment of bevacizumab. Under these analyses, we could infer that the tumor of the patient presented here might be generated from stem cells located close to SVZ; with the help of mutated PTEN genes and overexpression of VEGF, the tumor acquired highly progressive and metastatic features and showed characteristics of multifocal distribution. On the other hand, although 1p19q deletion is a well-known index for good prognosis, especially the sensitivity to temozolomide [28], this patient with 1p19q not deleted did show a positive response to chemotherapy.

The standard treatment for GBM includes surgery, temozolimide concomitant with radiotherapy, and followed by 6 cycles of adjuvant temozolomide. According to the results of EORTC trial EORTC-26981/CAN-NCIC- CE3, patients with GBM had a progression free survival (PFS) after 6 months of 53%, a median survival of 14.6 months, and a 2-year survival rate of 26.5% [1]. In fact, the best duration of adjuvant chemotherapy for patients with GBM is unknown. More and more clinicians started using temozolomide beyond 6 cycles. For example, Balañá *et al*. [29] mentioned that temozolomide treatment was continued for more than 6 cycles by 80.5% of neuro-oncologists in Spain. There are many kinds of long-term usage of temozolomide nowadays, three distinctive regimens are standard ‘5/28’ protocol (150 to 200 mg/m^2^ on days 1 to 5 every 28 days) [6], ‘1 week on-1 week off’ protocol (150 mg/m^2^ on days 1 to 7 and 15 to 21 of a 28-day cycle) [7], and dose-dense protocol (75 to 100 mg/m^2^ on days 1 to 21 every 28 days) [30].

For the presented patient, we chose the ‘5/28’ protocol: in addition to the standard 6 cycles, 6 more cycles of temozolomide (200 mg/m^2^) was added until recent clinical visit. After 8 cycles, significantly weakened signals in the frontal angle of left ventricle along with the pineal region on the Gd-enhance T1W image were seen, and after a 12-cycle chemotherapy, the enhancement of these two areas almost vanished. Epigenetic silencing by promoter methylation causes decreased MGMT expression and, thus, cannot maintain genomic integrity properly [31,32]. Many clinical researches found correlation between prognosis of patients with GBM and MGMT promoter methylation [32-34]. This patient had a methylated MGMT, and the response to extended temozolomide administration was well, which could prove that tailored plans for different patients due to their subtle pathological distinctions were necessary [35,36].

The patient tolerated this prolonged treatment well since no hematological or gastrointestinal toxicity occurred, and the tumor was controlled ideally. These results were consistent with many other clinical researches. Hau *et al*. [37] reported patients with primary GBM received a median of 13 cycles of temozolomide and had a time to progression (TTP) of 15 months and 2-year survival rates of 68%, and adverse events were thrombocytopenia (10%), leukopenia (7%), gastrointestinal toxicity (5%), and infection (4%). Seiz *et al*. [9] found that TTP and overall survival (OS) directly correlated with the amount of therapy, since there was a significant improvement of OS of patients who received more than 6 cycles of temozolomide compared to those who did not; by the way, moderate thrombocytopenia and pancytopenia occurred in 10% of all patients. Urgoiti *et al*. [8] reported a median survival of 24.6 months with minimal toxicity of patients who had more than 6 cycles, rather than 16.5 months for those stopped at cycle 6. Darlix *et al*. [6] also reported improvement of OS and PFS for patients with GBM who received at least 9 cycles (median = 14) of temozolomide than those who received 6 cycles (3-year PFS: 43.5% *vs*. 11%, 3-year OS: 48% *vs*. 22%).

There are still some limitations of our research. One is that exact pathologic study of every lesion seen in the MRI was not completely obtained. Since this patient has rapidly progressed and significant clinical manifestation was seen at the first view, we decided to resect the responsible lesion located in the right thalamus. And because the other lesions are diffusely located, one in the pineal region and the other in the frontal angle of the left lateral ventricle, we thought that multiple resections or biopsies might lead to a worse prognosis and delay the proper time of radiotherapy. Thus we did not have all lesions on the MRI under pathologic scan.

And also there might be other factors that help the patient have a good prognosis. Firstly, the patient has been under an operation which has released a lot of burden on the tumor. Secondly, this is a young patient with good tolerance to the possible side effects of long-term usage of temozolomide. Thirdly, this patient’s health insurance could cover the high expenses of 12-month temozolomide treatment, which has given the financial support for the patient and his family. Other patients with similar tumor characteristics of this one have been reviewed. Further data is under analysis.

## Conclusions

In summary, we propose that for patients with multifocal deep-seated glioblastoma multiforme, the resection of dominant lesion is the first and most fundamental step. Thorough pathologic examination for infiltrative and proliferative markers is necessary. Long-term temozolomide administration following the standard therapy might be a good choice for this kind of refractory tumor.

## Consent

Written informed consent was obtained from the patient for publication of this case report and any accompanying images. A copy of the written consent is available for review by the editor-in-chief of this journal.

## Abbreviations

GBM: glioblastoma multiforme
Gd: gadolinium
H-E: hematoxylin-eosin
KPS: Karnofsky Performance Scale
MGMT: O-6-methylguanine DNA methyltransferase
OS: overall survival
PFS: progression free survival
PTEN: phosphatase and tensin homolog
SVZ: subventricular zone
TTP: time to progression
VEGF: vascular endothelial growth factor
